# PyTrack: An end-to-end analysis toolkit for eye tracking

**DOI:** 10.3758/s13428-020-01392-6

**Published:** 2020-06-04

**Authors:** Upamanyu Ghose, Arvind A. Srinivasan, W. Paul Boyce, Hong Xu, Eng Siong Chng

**Affiliations:** 1 School of Computer Science and Engineering, Nanyang Technological University, Singapore, Singapore; 2 Present Address: Department of Computer Science, University of Oxford, Oxford, UK; 3 Psychology, School of Social Sciences, Nanyang Technological University, Singapore, Singapore

**Keywords:** Eye tracking, Software, Open source, Python

## Abstract

Eye tracking is a widely used tool for behavioral research in the field of psychology. With technological advancement, we now have specialized eye-tracking devices that offer high sampling rates, up to 2000 Hz, and allow for measuring eye movements with high accuracy. They also offer high spatial resolution, which enables the recording of very small movements, like drifts and microsaccades. Features and parameters of interest that characterize eye movements need to be algorithmically extracted from raw data as most eye trackers identify only basic parameters, such as blinks, fixations, and saccades. Eye-tracking experiments may investigate eye movement behavior in different groups of participants and in varying stimuli conditions. Hence, the analysis stage of such experiments typically involves two phases, (i) extraction of parameters of interest and (ii) statistical analysis between different participants or stimuli conditions using these parameters. Furthermore, the datasets collected in these experiments are usually very large in size, owing to the high temporal resolution of the eye trackers, and hence would benefit from an automated analysis toolkit. In this work, we present PyTrack, an end-to-end open-source solution for the analysis and visualization of eye-tracking data. It can be used to extract parameters of interest, generate and visualize a variety of gaze plots from raw eye-tracking data, and conduct statistical analysis between stimuli conditions and subject groups.

**Electronic supplementary material:**

The online version of this article (10.3758/s13428-020-01392-6) contains supplementary material, which is available to authorized users.

## Introduction

Relatively recent advancements in computer science and related technologies have resulted in greater automation and increased efficiency in analyzing large datasets when compared to manual processes. The field of psychology has greatly benefited from this in the form of advanced computational methods and tools. However, most of these software and tools are expensive and not freely accessible to everyone. Automating data processing with simple but effective computational pipelines provides the benefit of quicker and easier analyses. In turn, this allows more time for greater emphasis to be placed on designing paradigms, executing experiments, and arriving at the rationale for analyses.

While psychology is a broad discipline, a growing area of research is the analysis of eye movement and behavior as a tool for psychological insight. More specifically, eye tracking offers several interesting metrics, which can be beneficial for areas such as game design, visual marketing, medicine, and behavioral research like human emotion and deception detection. Some of the most important metrics, or parameters, include blinks, saccades, fixations, and pupil size. Each of these can act as proxies for different behavioral and physical conditions. For example, pupil dilation is known to be an indicator of stress (Pedrotti et al., 2014; Ren et al., 2014), while blink rate has been used to identify fatigue (Stern, Boyer, & Schroeder, 1994) as well as medical conditions such as schizophrenia (Chan & Chen, 2004). Eye tracking is also utilized in areas such as visual marketing (Wedel & Pieters, 2008) to gain insight into consumer behavior while searching for products in supermarkets or shops, and for gaining insight into user interaction with websites (Granka, Joachims, & Gay, 2004) in order to ameliorate user interface (UI) and user experience (UX) design for an improved experience. In the domain of behavioral research, eye tracking has proven to be a useful tool in the areas of human emotion and arousal analysis (Bradley, Miccoli, Escrig, & Lang, 2008) and has recently gained traction as a tool for deception detection (Cook et al., 2012; Kircher, 2018; Vrij, Oliveira, Hammond, & Ehrlichman, 2015). Considering its widespread influence over myriad domains, an open-source and freely accessible automated pipeline for parameter extraction and comparative analysis would be a beneficial addition to the eye-tracking community.

Here, we present PyTrack, an end-to-end analysis toolkit, built using the Python programming language, that allows users to analyze eye-tracking datasets using a few lines of code, as shown in a sample segment in Listing 1. After the initial process of recording eye movement data for multiple participants in multiple stimulus conditions, the raw data exported from the eye tracker can be directly fed into PyTrack in order to perform parameter extraction, generate plots and conduct statistical analysis on the extracted parameters. The toolkit can generate gaze plots, gaze heat maps, dynamic pupil and gaze plots, and aggregate heat maps for a group of participants. PyTrack also extracts parameters related to pupil size, blinks, fixations, saccades, microsaccades, and reading behavior. We have also implemented a feature that allows the user to indicate an area of interest (AOI) for the stimuli, in order to extract more advanced parameters such as number of revisits. If the experiment involves different groups of participants or stimulus conditions, PyTrack also provides the functionality of performing statistical tests such as the *t* test and variants of ANOVA for combinations of between and within group parameters. However, if desired, PyTrack can export a formatted file containing the extracted parameters without performing any statistical analysis, in order to allow the users to perform their own analyses. This facilitates a high degree of flexibility for the end-user in terms of analytical requirements because it can easily be used for rapid automated end-to-end analysis of eye-tracking experiments or simply as a parameter extraction tool. The programming required to utilize most features of PyTrack is minimal and can be accomplished by adapting parameters of the sample code segments. The visualization component has a simple graphic user interface (GUI) for ease of navigation and selection of participants and stimuli. For the more advanced and experienced programmers, PyTrack's functions can also be used and modified by importing it as a Python library. We discuss the toolkit in greater detail in sections ‘Framework Structure’ and ‘Features of PyTrack’.

**Listing 1.**
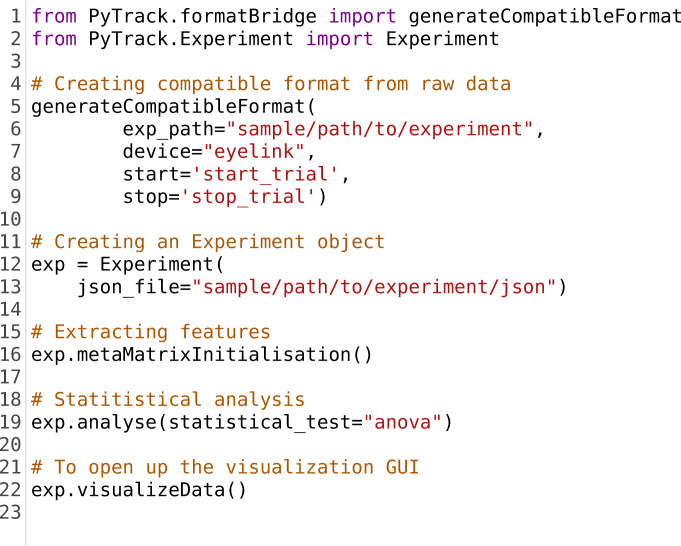
Sample code segment to use PyTrack. **Line 5:** Using the format bridge to convert data into the compatible format. **Line 12:** Creating the main Experiment class object to perform analysis and visualization. **Line 16 and 19:** Extracting the metadata or parameters and performing the ANOVA test between subject or participant groups. **Line 22:** Invoking the visualization GUI to view and save the various plots

### Related work

Most existing software and libraries for eye-tracking analysis are hardware specific proprietary software that is provided with the eye-tracking device. However, there have also been some significant contributions by individuals to the open-source community of eye-tracking research.

Among the hardware specific proprietary software, the first we will discuss is the SMI BeGaze™ (Sensomotoric Instruments, 2016) analysis suite. It supports various functionalities such as semantic gaze mapping, heat map generation, fixation mapping, area of interest (AOI) analysis, and extraction of key performance indicators such as number of revisits to AOI. Additionally, it provides (i) duration and velocity dispersion-based event detection for saccades and fixations, and (ii) extraction and exporting of several parameters related to saccades, fixations, blinks, and pupil size. Any reference to ‘events’ from here on will refer to fixations, saccades and blinks. Tobii provides its own software called Tobii Pro Lab™ (Tobii Technology, 2019) for design of experiments as well as analysis of collected data. It supports similar functionalities to BeGaze™ such as heat map and gaze plot visualization, area of interest analysis, and extraction of events. SR Research provides the Data Viewer™ (SR Research, 2018) software for EyeLink devices. It is mainly used for visualizing gaze and pupil-size data. It also allows event detection and generation of aggregate event and AOI reports and there is extended support available via MATLAB®, Psychtoolbox ® and E-Prime ®. However, what is common among these software programs is that they must be purchased in order to access the advanced functionalities. Of particular note is that the above software does not facilitate the extraction of microsaccade parameters from fixations and do not support multiple data formats from other hardware.

Other than the software accompanying eye-tracking hardware, there are some proprietary software such as iMotions™ (iMotions, 2019) that act as presentation, data collection and analysis software. iMotions™ provides several analysis tools that allow parameter extraction from raw data after the process of data collection. For example, iMotions™ supports aggregate heat map generation, fixation plot generation and AOI analysis including totaling number of revisits, time spent etc. It also has the provision of the dynamic viewing of pupil size, eye distance and gaze data. However, it is an expensive software and, at the time of writing, does not support some advanced eye trackers such as the EyeLink 1000 Plus.

Among the open-source eye-tracking tools, PyGaze (Dalmaijer, Mathôt, & Van der Stigchel, 2014) is a commonly used library. Its main functionality is enabling recording of eye-tracking data using presentation software such as OpenSesame (Mathôt, Schreij, & Theeuwes, 2012). It facilitates the sending of triggers and messages. It also provides some basic analysis functionality such as event detection, heat map generation and fixation plot generation. However, it does not provide any advanced parameter extraction, aggregate experiment analysis, or statistical tests. As its main functionality is to act as a wrapper around several existing packages, it does not provide a lot of functionality for post recording analysis. Another open-source package available for analysis in the R programming language is eyetrackingR (Dink & Ferguson, 2015). It supports most of the widely used eye trackers such as EyeLink and Tobii. The functionalities it provides are ‘cleaning-up’ and performing different analyses on time series data such as growth-curve analysis and onset-contingent reaction time analysis. It also provides the functionality of generating plots corresponding to the analyses. However, it does not provide support for the extraction of parameters related to fixations, saccades, microsaccades, pupillometry, and blinks. A similar package for Matlab® is the EMA Toolbox (Gibaldi & Sabatini, n.d.). It supports EyeLink, SMI, and Tobii eye trackers. The functionality provided by EMA includes: data conversion from normalized to pixel to degrees; saccade identification; saccade kinematics; generation of saliency maps; and generation of main sequence plots. Main sequence is a plot that shows the relationship between the peak velocity (on the vertical axis) and amplitude (on the horizontal axis) of saccadic eye movements. OGAMA – Open Gaze and Mouse Analyzer (Voßkühler, Nordmeier, Kuchinke, & Jacobs, 2008) is another open-source software which is similar to the iMotions™ software discussed earlier. It allows creation and presentation of stimuli, recording of eye-tracking data and finally, analysis and visualization of the collected data. It provides the functions of dynamic replay of data, generation of fixation path plots, and attention maps. Users can specify subject, trial, gaze, and mouse event parameters that OGAMA calculates and then exports into a results file. The OGAMA software only supports extraction of parameters related to fixations and saccades.

### Framework structure

The PyTrack framework is designed using principles of object-oriented programming (OOP) and is structured in a manner that provides maximum flexibility to the user. The first design component takes into consideration the users' varying knowledge of Python programming. We understand that users require different levels of access to, and flexibility with, the framework, depending upon their programming knowledge. For users with limited programming experience, PyTrack can act as an end-to-end toolkit taking raw data as input, perform all the required parameter extractions and producing the results of statistical analysis as output. Advanced programmers, on the other hand, have the flexibility of accessing the extracted parameters, member functions and variables, and using them for their custom requirements.

**Fig. 1.**
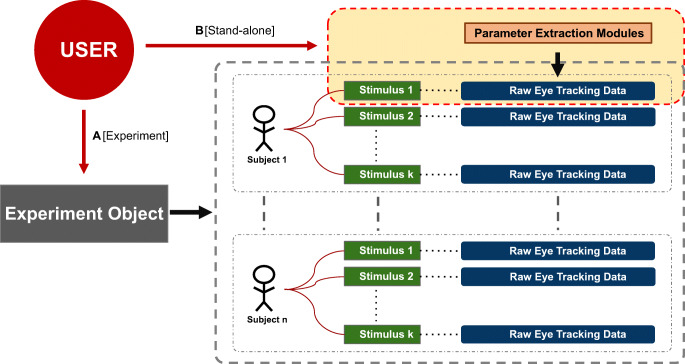
PyTrack framework structure. The structure is based on object-oriented programming concepts where different objects interact with each other. The “Experiment” object (*grey*) interacts with multiple “Subject” objects (*stick figure*). These in turn interact with multiple “Stimulus” objects (*green*) analogous to the stimuli presented to the subjects during the experiment. Each “Stimulus” object applies the parameter extraction modules (*orange*) to the corresponding raw eye-tracking data (*blue*). The path *A [Experiment]* from the *User* (*red*) shows the user-toolkit interaction in the experiment design mode as discussed in the section “Framework Structure". The path *B [Stand-alone]* from the *User* shows the stand-alone design structure where the user can work on a single subject at the individual stimulus level or just use the parameter extraction modules independently. The documentation (found here https://pytrack-ntu.rtfd.io) explains how to use PyTrack in each design mode

The second design component of PyTrack takes into consideration how a given user may wish to use PyTrack, either in the ‘experiment design’ or ‘stand-alone design’. This is achieved by the entire functionality being broken-up into different objects that interact with each other, as can be seen in Fig. 1.

In the experiment design, PyTrack can analyze an entire experiment containing *n* subjects responding to *k* stimuli each. Path ‘A [Experiment]’ in Fig. 1 illustrates the structure of this design. In this setup, the user interacts with the Experiment object and PyTrack effectively acts as a black box, thus removing any need of the user to consider internal functionalities. However, as the objects at different levels interact with each other, if the user wishes, the member variables and functions of the internal objects can also be accessed through the Experiment object. This, of course, would require programming knowledge in Python, as discussed earlier.

The stand-alone design is for those users who wish to analyze a single stimulus for a given subject or make use of the parameter extraction functions and visualization tools for their custom needs. The user–toolkit interaction can be seen in Path ‘B [Stand-alone]’ of Fig. 1. The user interacts directly with the internal objects and parameter extraction modules. Hence, this is designed for those who wish to access PyTrack at a lower level of the abstraction model to directly control the algorithms and visualization functionality.

### Features of PyTrack

In the following section we discuss the salient features of PyTrack, giving an overview of its robust functionality and how it acts as an end-to-end toolkit.

### Format agnostic and quick data access

The format bridge module in PyTrack enables the analysis and visualization of data collected using three different types of eye trackers - EyeLink, Tobii, and SMI. This functionality was achieved by adapting and modifying the code in PyGazeAnalyser (Dalmaijer et al., 2014). The recording software of the eye trackers provide the option of exporting the data as raw text files. The format bridge module accepts these files as its input and converts them to a base format readable by PyTrack, a comma separated value (CSV) file. Following this, an SQL database is generated containing an aggregation of all the participants. The rationale for generating an SQL database and using it to access the data instead of the CSV files is that it reduces the time required to read data when conditional querying is implemented. Working with raw SQL files is inconvenient and hence, we transform the SQL data into a Pandas DataFrame for internal usage.

We conducted data access tests on two different systems using datasets of varying number of rows in CSV and SQL formats. Table 1 shows the difference in data access times for each of the cases with and without conditional querying. With an increase in the number of rows, systems with better processors and higher random-access memory (RAM) are able to read the entire data much quicker. This is because a higher RAM allows more data from the SQL or CSV tables to be loaded into the main memory for quick access. This in turn leads to a lower number of page faults (data that need to be accessed but are not present in the main memory) and reduces the latency of accessing the secondary memory (hard disk, solid-state drive etc.). The SQL data access time shown is the cumulative time for accessing the raw SQL data in Python and to convert it to a Pandas DataFrame. The benefit of using Pandas is that it allows easy and efficient manipulation of SQL and CSV data in Python. As can be seen, if all the stimuli from an experiment are being analyzed, the CSV reading option is the faster approach. However, if only a subset of stimuli is being analyzed which involves conditional data access, SQL provides a significant decrease in access time. Therefore, depending on the task, the user may toggle the access method from CSV to SQL or vice-versa.

**Table 1.** Data access times (CSV vs. SQL)

System configuration (RAM and CPU)	Number of rows	CSV (ms)	SQL (ms)	CSV with condition clause (ms)	SQL with condition clause (ms)
RAM: 4 GB CPU: Intel i7-5500U	10,000	26.86	46.67	45.66	17.80
	100,000	250.24	455.61	380.89	185.05
	1,000,000	2463.14	4558.18	3773.78	1732.73
RAM: 32 GB CPU: Intel i7-8700	10,000	18.62	40.37	44.97	16.80
	100,000	185.61	335.48	229.43	119.04
	1,000,000	1795.01	3578.18	2230.54	1176.60

### Parameter extraction

PyTrack extracts 21 parameters in all, which are commonly used in eye-tracking research. These comprise pupil size, blinks, fixations, saccades, microsaccades and reading behavior.

#### Pupil size

Pupil size, and parameters derived from it, have been used to study the dynamics of cognitive brain functions (Beatty, 1982). Research suggests that changes in pupil size can be used as an index for attentional effort (Kang, Huffer, & Wheatley, 2014) and non-emotional perceptual tasks (Webb, Honts, Kircher, Bernhardt, & Cook, 2009). The pupil size parameters that are provided by PyTrack are: average pupil size; time to pupil size peak; peak pupil size; and area under the pupil curve.

#### Blinks

Parameters related to blinking are used as proxies to medical conditions and fatigue as mentioned in ‘Introduction’. Blinks can easily be detected from eye-tracking data by finding segments of the raw data where the pupil size falls to zero. However, Hershman, Henik, and Cohen (2018) proposed a noise-based blink detection algorithm that detects the onset and offset of the blinks more accurately. Hence, we adapted their algorithm to implement a python version and validated it with the original implementations in R and MATLAB. The comparison of the two implementations is shown in Supplementary Fig. 1. The first step of the algorithm is to identify the missing values in the pupil size data and mark the last valid sample before and after the missing segment as the initial blink onset (sample *n*) and offset (sample *m*). The pupillometry data is then smoothened using a moving average filter with a window size of 10 ms and the difference between adjacent samples is calculated. For example, if *p*_*t*_ (*t* =1, 2, 3, 4, 5, …) is the pupil size at any time *t*, the values calculated are *p*_*2*_ – *p*_*1*_, *p*_*3*_ – *p*_*2*_, *p*_*4*_ – *p*_*3*_*,* and so on. This difference is calculated in order to identify monotonic sequences in the smoothened pupillometry data, which in turn is used to update the old blink onset and offset (sample *n* and *m*, respectively). The blink onset is updated by starting at the initial onset (sample *n*) and moving backward (sample *n-1*, *n-2*, …) while the pattern is monotonically increasing. The index of the last value in this pattern before the initial onset is selected as the new blink onset. Similarly, for the blink offset, we move forward from the initial offset (from sample *m* to *m+1*, *m+2*, …) while the pattern is monotonically increasing and select the last value in the pattern after the initial offset as the new blink offset. The blink parameters that PyTrack provides are: blink count; peak blink duration; and average blink duration.

#### Fixations

The duration and rate of fixations have been used in the past for studying deception by Cook et al. (2012), and cognitive process by Just and Carpenter (1976). Furthermore, the ratio of fixation count and duration inside and outside an AOI can also help in determining the level of focus of the subject in the given AOI, which is of importance in a variety of tasks such as visual marketing research and reading research (Daneman & Reingold, 1993; Just & Carpenter, 1980). The fixation sequences are obtained from the raw data exported from the eye tracker’s software. In cases where the data does not contain this information, the dispersion-based threshold identification (I-DT) algorithm is applied to the gaze data in order to identify the fixations (Salvucci & Goldberg, 2000). The first step is selecting a window within which the dispersion *D* is to be calculated. We consider a window size *W*_thresh_ of 50 ms as the minimum duration for a segment to be classified as a fixation. Starting with the minimum window size i.e. *W* = *W*_thresh_, the dispersion is calculated using Eq. (1). In the equation, *G*_*x*_ and *G*_*y*_ are the gaze position vectors in the *x* and *y* axes for a given window and *D* is the dispersion of that window. If the *D* <= *D*_thresh_, the window size is increased by 1, i.e., *W* = *W*+1, and the previous step is repeated. Finally, if *D* > *D*_thresh_ and *W* > *W*_thresh_, the points in the window excluding the last point are considered to be part of a fixation sequence. Then the window resets to *W*_thresh_ and starts at the first point after the last fixation sequence.

1 $$ D=\sqrt{\left({\left[\max \left({G}_x\right)-\min \left({G}_x\right)\right]}^2+{\left[\max \left({G}_y\right)-\min \left({G}_y\right)\right]}^2\right)} $$

After this, for a given stimulus, PyTrack extracts the fixation count, maximum duration and average duration. In order to get fixation parameter values inside an AOI, the area can be specified before analysis, as explained in ‘area of interest (AOI)’.

#### Saccades and microsasccades

Saccade sequences are usually marked by the eye tracker’s recording software in the raw data. For data in which the saccade sequences are not marked, the velocity-based threshold identification (I-VT) algorithm is applied (Salvucci & Goldberg, 2000). The first step in the algorithm is to calculate the pointwise velocity of the gaze data. Points with velocities of more than around 40 pixels/second are classified as saccades. Equation (2) shows the computation of the velocities from gaze data. In the equation, *V*_*t*_ is the velocity in *x* or *y* direction at time *t,* and *G*_*t*_ is the gaze position in *x* or *y* axis at time *t*.

2 $$ {V}_t={G}_t-{G}_{t-1} $$

A well-known and widely used algorithm for the detection of microsaccades was first proposed by Engbert and Kliegl (2003) followed by improvements made by Engbert and Mergenthaler (2006). In PyTrack, we provide an implementation of this algorithm in order to extract parameters such as count, duration, velocity, and amplitude. The first step is to calculate the velocity from the gaze data using a moving average , as shown in Equation (3a). The next step is the calculation of the velocity threshold *V*_thresh_ using Equation (3b). The *k* value is calculated using Equation (3c), and all points with *k* > 1 are considered to be part of a microsaccade sequence, provided the sequence contains at least six samples. Finally, the velocity and amplitude of the microsaccade sequence are calculated using Equation (3d & e). The last two equations are also used to calculate the velocity and amplitude of the saccades. In Equation (3): *V*_*t*_ is the velocity in *x* or *y* axis at time *t*; *G*_*t*_ is the gaze position in *x* or *y* axis at time *t*; *S*_freq_ is the sampling frequency of the eye tracker; *V*_thresh_ is the microsaccade threshold velocity in the *x* or *y* axis; *V*_fac_ is a constant value that is used to calculate the microsaccade threshold velocity in the *x* or *y* axis (default value used is 5); *V*^→^ is the velocity vector in the *x* or *y* axis; *G*^→^ is the gaze position vector in the *x* or *y* axis; *V*_peak_ is the peak velocity of the microsaccade sequence; and *A* is the microsaccade sequence amplitude.

3a $$ {V}_t=\left({G}_{t+2}+{G}_{t+1}-{G}_{t-1}-{G}_{t-2}\right)/\left(6\times {S}_{\mathrm{freq}}\right)\kern0.5em $$

3b $$ {V}_{\mathrm{thresh}}={V}_{\mathrm{fac}}\times \sqrt{median\left({\left(\overrightarrow{V}- median\left(\overrightarrow{V}\right)\right)}^2\right)}\kern0.75em $$

3c $$ k={\left(\overrightarrow{V_x}/{V}_{x_{\mathrm{thresh}}}\right)}^2+{\left(\overrightarrow{V_y}/{V}_{y_{\mathrm{thresh}}}\right)}^2\kern0.5em $$

3d $$ {V}_{peak}=\max \left(\sqrt{{\overrightarrow{V_x}}^2+{\overrightarrow{V_y}}^2}\right)\kern0.5em $$

3e $$ A=\sqrt{{\left[\max \left(\ \overrightarrow{G_x}\right)-\min \left(\ \overrightarrow{G_x}\right)\right]}^2+{\left[\max \left(\ \overrightarrow{G_y}\right)-\min \left(\ \overrightarrow{G_y}\right)\right]}^2}\kern0.5em $$

In order to ensure that our implementation is in accordance with the original algorithm adapted for R, we compared the output plots on the same data file. The plot in Supplementary Fig. 2a shows the microsaccade gaze position and gaze velocity plots generated by PyTrack. Supplementary Fig. 2b shows the same plots generated by the MS Toolbox for R (Ralf Engbert, Mergenthaler, Sinn, & Pikovsky, 2011; Ralf Engbert, Sinn, Mergenthaler, & Trukenbrod, 2015) as an implementation of their own algorithm. A comparison of the two plots shows that both implementations work identically.

#### Reading behavior

PyTrack also extracts parameters to look at reading behavior of subjects. Cook et al., (Cook et al., 2012) saw that, during deception detection, reading patterns could be used for differentiating between participants assigned to an ‘innocent’ group and participants assigned to a ‘guilty’ group. One reading of the text located in the AOI is defined as the set of consecutive fixations which are located in the specified region. The reading parameters included in PyTrack are: number of readings; duration of first reading (first pass); and duration of second reading (second pass).

### Area of interest (AOI)

**Fig. 2.**
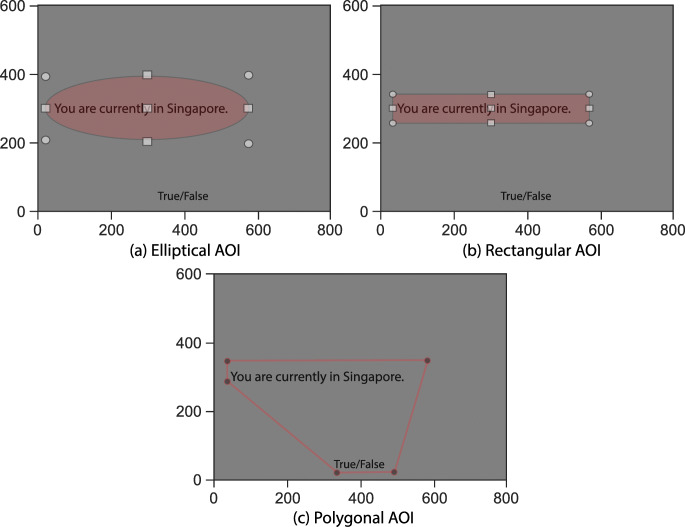
Different AOI shapes supported by PyTrack

PyTrack facilitates user specification of an AOI from which parameters of interest can be extracted. If there is a common AOI for all stimuli, it can be drawn or specified as coordinates. The accepted drawn shapes are rectangles, ellipses, and polygons. If the coordinates are specified, they can be one of the following shapes: the top-left and bottom-right coordinates of a rectangle, center and size of axes (width and height) of an ellipse, or the coordinates of a polygon's vertices. However, if the AOI is different for each stimulus, the values must be specified as rectangle, ellipse, or polygon coordinates in a CSV file, along with the corresponding stimulus name. The drawing functionality is not supported in this case because the task of drawing an AOI for all the stimuli is rather tedious and as the AOIs are usually defined at the time of conducting the experiment, it is more convenient to specify them in a CSV file. Several experiment presentations and recording software such as OpenSesame allow exporting such parameters as CSV files. Figure 2 shows the different types of AOI PyTrack accepts.

### Statistical analysis

The analysis stage of experiments usually involves the comparison of (i) a subject or subject group’s response to different types or classes of stimuli and (ii) the response of different subject groups to the same stimulus type. PyTrack has inbuilt statistical analysis functions (e.g., ANOVA, mixed ANOVA, *t* test, etc.) that allows the users to conduct a variety of tests to compare the various subject and stimuli groups. These tests provide statistical analyses of the extracted parameters discussed earlier. The users can either conduct the tests on all the extracted parameters, or alternatively specify the parameters of interest and test for statistically significant differences on the specified parameters. Lines 9 and 13 in Listing 2, respectively, contain sample code segments to execute these functionalities.

**Fig. 4.**
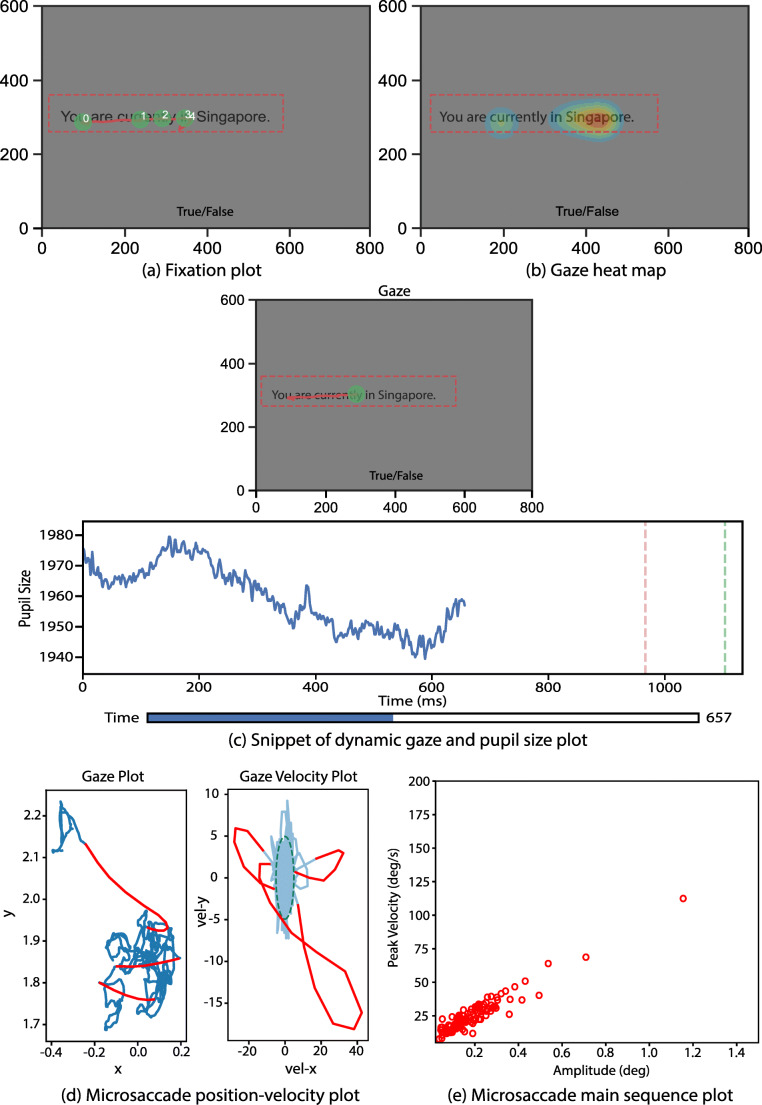
Plots generated by PyTrack visualization. (**a**) Fixation plot with the fixations marked with green circles and numbered in order of occurrence. (**b**) Gaze heat map illustrating the regions of the stimuli most viewed by the subject. (**c**) Snippet of the dynamic pupil size plot which shows the change in pupil size as the subject views the stimulus. (**d**) Microsaccade position-velocity plot for all microsaccades within a single fixation for a given stimulus. (**e**) Microsaccade main sequence plot for all microsaccades within all fixations for a given stimulus

A basic test that is supported is a mixed ANOVA, which considers stimuli type to be a within group factor and subject type as the between group factor. In addition to this, as shown in line 17 of Listing 2, PyTrack also allows for advanced analysis where the user can specify additional within and between group factors on which statistical analysis can be performed. It provides for *n*-way ANOVA, repeated measures ANOVA (RMANOVA), pairwise Student’s *t* test and pairwise Welch *t* test. The *n*-way ANOVA accepts any number of between group factors while the RMANOVA accepts up to two within group factors. The pairwise Student’s *t* test can be used when analysis is conducted for the within-subject factor, in within-subject designs (one-way) or mixed designs (with one between group and one within-group factor). The Welch *t* test accepts only one within group or one between group factor. The Mixed ANOVA, RMANOVA, and pairwise *t* test are performed by using the functions defined in the pingouin package (Vallat, 2018), while statsmodels (Seabold & Perktold, 2010) is used for *n*-way ANOVA. Scipy (Oliphant, 2007) provides the functionality to perform the Welch *t* test.

Users can perform analysis of a select few parameters, instead of all of them, by specifying the parameters of interest. The results of these tests are printed out in the computer terminal and saved into CSV files for later reference. PyTrack also provides the option of allowing users to export all the extracted parameters in the form of a CSV file without performing any tests, as shown in line 23 of Listing 2. The data present in the CSV file contains all the extracted parameters along with the name of the subject and stimuli that was being observed. This is especially useful if the users wish to perform statistical tests independent of PyTrack.

### Ease of modifying analysis factors

The analysis of the entire experiment is controlled by an intuitive JSON file. Users can easily specify the list of participants and stimuli to be analyzed. Participants and stimuli can also be grouped as required. They also can be added or removed from the JSON file in order to modify the analysis and focus on a subset of the entire corpus. Additionally, every participant or stimulus can be assigned a list of attributes such as gender and age for participants, and brightness level for stimuli. This enables the users to apply the statistical tests to more than one between group factor. To form a baseline for comparisons, the names of control stimuli can also be specified in PyTrack. The parameters extracted from these are used to normalize the parameters extracted from the rest of the stimuli. Parameters relevant to the hardware used in the experiment such as display screen dimensions and sampling frequency of the eye tracker can also be specified here. Essentially, all the parameters involved with analyzing the experiment data can be specified in a single file, thereby simplifying and speeding up the process of analysis. The structure of this JSON file can be seen in Listing 3. The users need not write this file from scratch as a template can be downloaded as part of the sample data.

**Listing 3.**
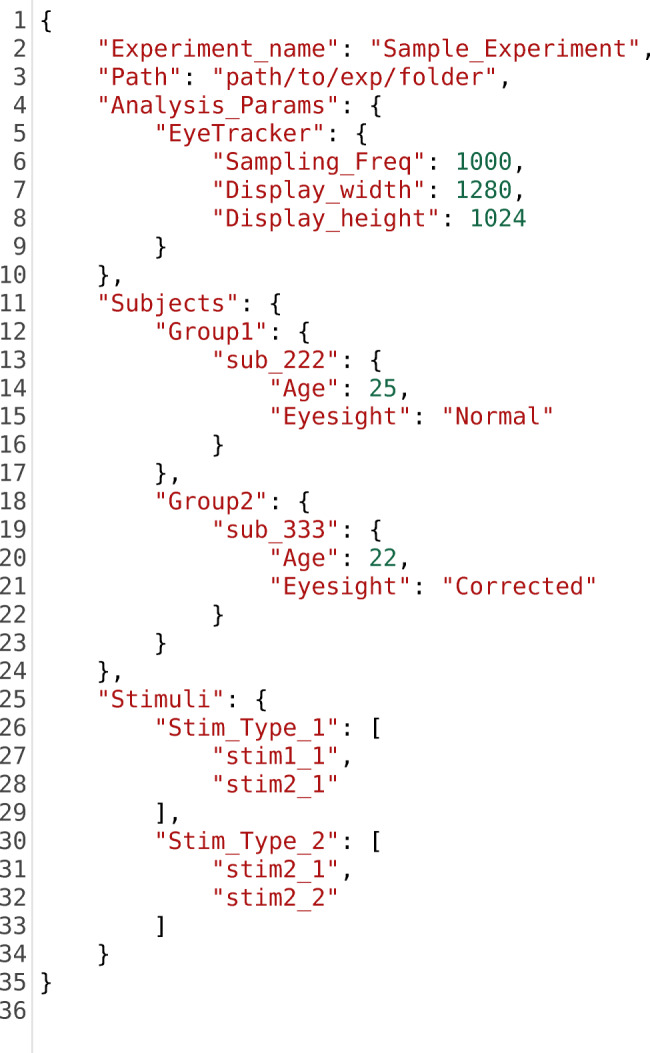
Sample Experiment JSON file structure. There are two subject groups – Group 1 and Group 2. Each subject has Age and Eyesight information associated with them which may be used as additional between group factors while conducting statistical tests. There are two stimuli groups – Stim_Type_1 and Stim_Type_2. Although there are only two groups of subject and stimulus in this example, users may specify one or more groups

### Visualization

**Fig. 3.**
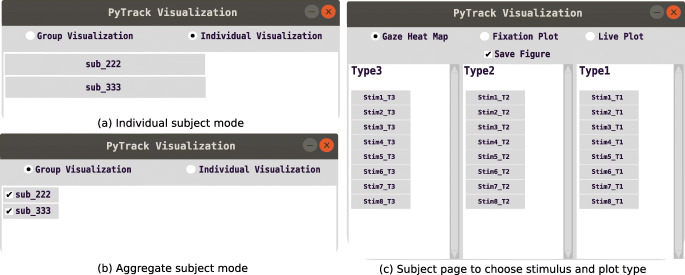
Sample GUI windows in PyTrack visualization. (**a**) The individual subject window allows the user to choose a specific subject and view the various plots of that subject for the various stimuli. (**b**) The aggregate or group subject window allows the user to select multiple subjects simultaneously and view the plots of the selected group for various stimuli. (**c**) This page is for the user to specify the plot type – fixation, gaze heat map, or dynamic pupil size – and the stimulus for which the plot is desired. There is also an option to save the plots

Another set of important features of PyTrack are the visualization tools it provides. These vary based on the mode PyTrack is being used in, as mentioned in ‘Framework Structure’. The interaction in the Experiment Design is via a simple GUI that allows easy and convenient navigation with two broad categories—individual subject and aggregate. The visualization windows are shown in Fig. 3. In the Stand-alone Design, the visualization is for the individual stimulus. Function invocations by code can generate the same plots as in the Experiment Design, with the exception of the aggregated subject plots.

The individual subject category includes fixation plots, gaze heat maps and dynamic gaze, and pupil size plots. The fixation plot, as shown in Fig. 4a, is a gaze plot with numbered fixation points which enables the user to view the order in which the subject viewed the stimulus and what regions they fixated on. Fig. 4b shows the gaze heat map, which is essentially a smoothed two-dimensional histogram of the subject's gaze data. It is a visual representation of the frequency with which regions of the stimulus were viewed. The gaze coordinates of the subject, over time *t,* are taken and the ‘histogram2d’ function of NumPy (Oliphant & Millma, 2006) is applied. If the number of rows and columns of pixels in the original image are *n*_rows_ and *n*_cols_*,* the number of histogram bins assigned are (*n*_rows_*)*/4 and (*n*_cols_*)*/4. This is followed by a Gaussian filter that smooths the histogram, and a contour plot of the filtered 2D histogram is generated using an appropriate color map to produce the final heat map. The last type of plot is the dynamic pupil and gaze plot, which is shown in Fig. 4c. It is a dynamic (moving) plot of the gaze on the stimulus along with the corresponding pupil size of the subject at that time instance. As the navigation is via a GUI, it is convenient to close a particular plot and generate another one for the same subject or a different subject. Furthermore, there is an option to save the plots generated into the experiment folder which can be accessed at a later time as needed.

**Listing 2.**
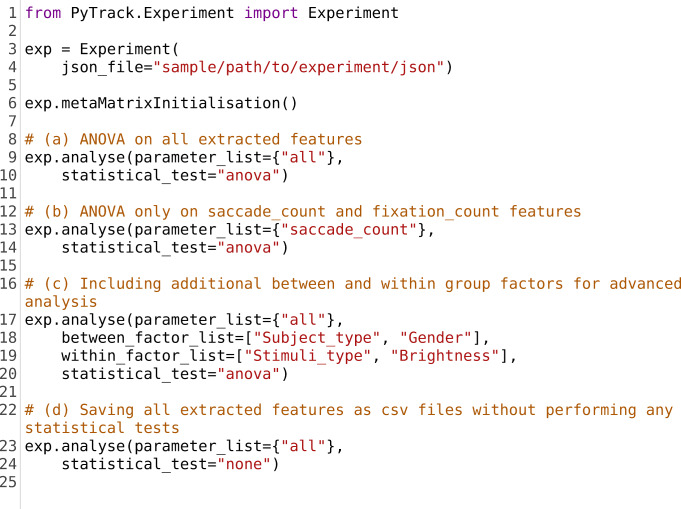
Sample code segment to run different statistical tests in PyTrack. For all tests, the default between group is subject or participant type. **Lines 9–10**: Function to run the ANOVA test between subject groups on all extracted parameters. **Lines 13–14**: Function to run the ANOVA test between subject groups only on saccade_count. **Lines 17–20**: Function specifying gender as the additional between group factor and brightness as the additional within group factor. It runs the ANOVA test for the specified between and within group factors on all extracted parameters. **Lines 23–24**: Function to export all the extracted parameters in a formatted CSV file without running any statistical tests

The aggregated subject plot is available if used in the Experiment Design and shows aggregated heat maps for a given stimulus. The user has the freedom to select the subjects that are required for the aggregate analysis and if desired the plots can be saved along with a text document containing the list of subjects included in that plot. Aggregated heat maps act as a visual representation of the average time spent by all the subjects in viewing the various regions of a stimulus. In cases where there are different groups of subjects, this may help provide an insight into the differences in viewing patterns between said groups.

Additional features available in the Stand-alone Design include the generation of microsaccade position and velocity plots and main sequence plots. The main sequence, in this case, is the plot between the peak velocity and amplitude of the microsaccades. These can be generated in the Experiment Design also, but as it is an advanced feature it requires the user to add an additional line of code. Some samples of the plots generated are shown in Fig. 4d and Fig. 4e.

### Using PyTrack as a parameter extraction tool

**Fig. 5.**
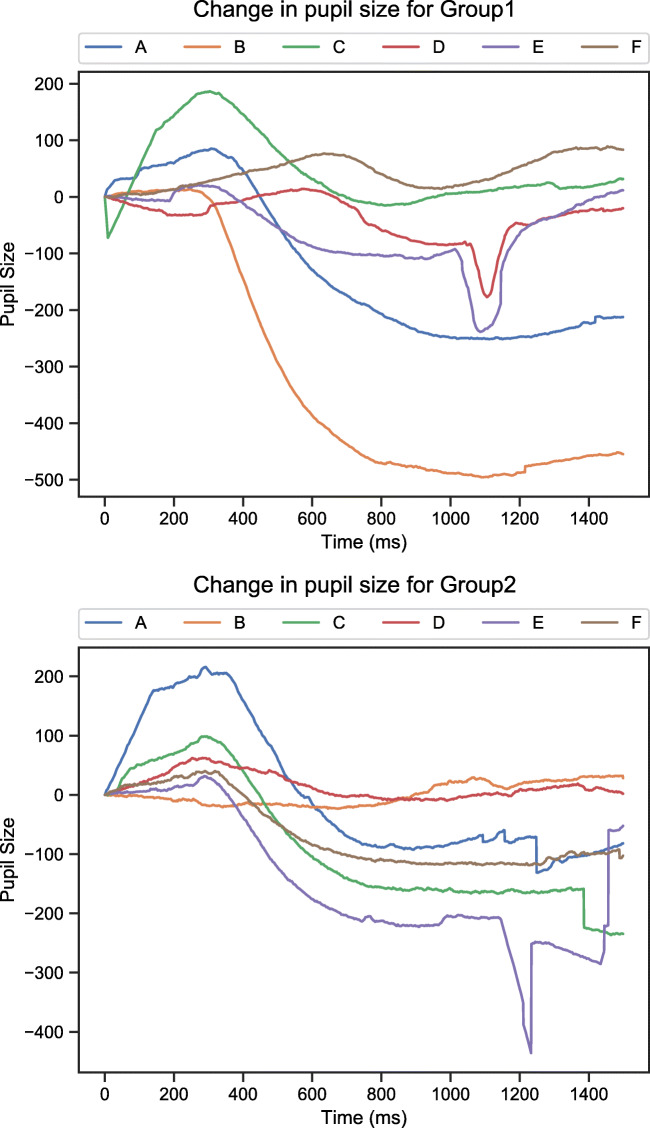
Pupil size plots for each subject group (Group 1 and Group 2) showing the difference between mean interpolated pupil size for each of the stimulus conditions (A, B, C, D, E and F). The plot was generated by Listing 4 using the sample data provided in the NTU_Experiment folder (https://osf.io/f9mey/files/)

PyTrack can also be used just as a parameter extraction tool instead of an end-to-end solution. The parameters extracted can then be used to generate custom plots or perform custom analyses as desired by the users. Listing 4 contains a code segment showing one possible use of PyTrack as an extraction tool. The code segment generates pupil size plots, comparing various stimulus conditions for each participant or subject group. Lines 15–28 in the code listing demonstrate how the extracted parameters can be accessed using Python code. The example parameter accessed in the listing is *InterpPupilSize,* which stands for interpolated pupil size, and demonstrates the generation of comparative pupil size plots*.* All parameters can be accessed in a similar fashion. Lines 35–45 contain the code to generate the pupil size plots mentioned earlier. This can be replaced by the users’ code to generate custom plots or perform their own analyses. The principle behind accessing the extracted parameters remains the same. Figure 5 shows the plots generated by this code listing using the provided sample data.

**Listing 4.**
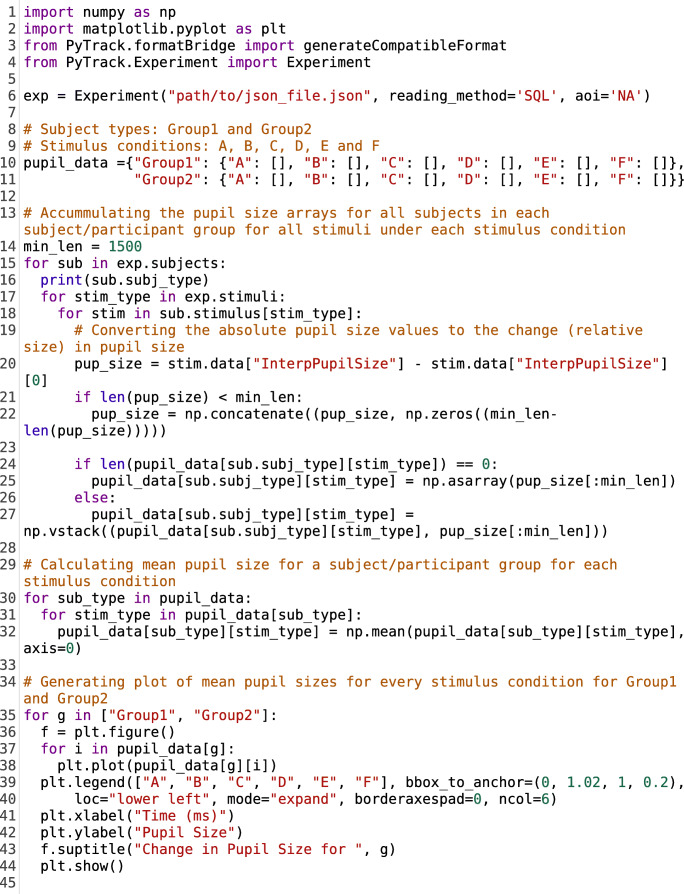
Sample code segment showing the use of PyTrack as a parameter extraction tool. In this case, PyTrack is being used to generate comparative pupil size plots. **Lines 15–28:** Looping through all stimuli (in every stimulus condition) for each subject (in all the subject groups) and accumulating the interpolated pupil size. **Lines 30–32:** Finding the mean interpolated pupil size for each stimulus condition for the different subject groups. **Lines 35–44:** Plotting the mean interpolated pupil size data for every stimulus condition for subject groups 1 and 2

### Comparison

PyTrack offers the same essential functionality present in the existing toolkits and frameworks discussed in ‘Related Work’. However, there are several features that differentiate it from existing software: it has the added benefits of being format agnostic; it is an end-to-end solution with the addition of statistical analysis capabilities; it is open source; and it is free to use. The visualization interface is straight-forward and easy to navigate, and the analysis tools are easy to use. Furthermore, it can be used in different modes - Experiment and Stand-alone - and at various levels of abstraction based on the desired flexibility and modification.

A comparison with SMI BeGaze™, Tobii Pro Lab™, EyeLink Data Viewer™, iMotions™, PyGaze, eyetrackingR, EMA Toolbox and OGAMA can be seen in Table 2. There are certain areas in which PyTrack can be improved, as discussed in ‘Conclusions’, but several more in which it proves to be a beneficial tool when compared to similar software.

**Table 2.** Comparison of eye tracking analysis software

	SMI BeGaze	Tobii Pro Lab	EyeLink Data Viewer	iMotions	PyGaze	OGAMA	eyetrackingR	EMA Toolbox	**PyTrack**
Free to use	✗	✗	✗	✗	✓	✓	✓	✓	✓
Format agnostic	✗	✗	✗	✓	✓	✓	✓	✓	✓
Blink parameters	✓	✓	✓	✗	✓	✓	✗	✓	✓
Fixation parameters	✓	✓	✓	✓	✓	✓	✗	✓	✓
Saccade parameters	✓	✓	✓	✓	✓	✓	✗	✓	✓
Microsaccade parameters	✗	✗	✗	✗	✗	✗	✗	✗	✓
Different AOI shapes	✓	✓	✓	✓	✗	✓	✗	✗	✓
Multiple AOIs	✓	✓	✓	✓	✗	✓	✓	✗	✗
Dynamic AOIs	✓	✓	✓	✓	✗	✓	✓	✗	✗
Fixation plot	✓	✓	✓	✓	✓	✓	✗	✗	✓
Gaze heat map	✓	✓	✓	✓	✓	✓	✗	✓	✓
Dynamic gaze and pupil size plot	✓	✓	✓	✓	✗	✓	✗	✗	✓
Analysis GUI	✓	✓	✓	✓	✗	✓	✗	✓	✗
Visualization GUI	✓	✓	✓	✓	✗	✓	✗	✓	✓
End-to-end experiment analysis	✗	✗	✗	✗	✗	✗	✗	✗	✓

## Conclusions

Considering a trade-off between cost and ease of use, as outlined in Table 2, PyTrack is a promising option that is free-for-all. However, we believe that there is scope for including more advanced analysis methods, improving the user interface, and improving parameter detection algorithms with advances in eye-tracking research.

### Analysis methods

The methods provided are variants of the ANOVA and *t* test, which are purely statistical methods. There are several machine learning methods which can aid in further analysis and build classification models based on the experiment data. Therefore, it is possible to integrate these methods into the toolkit which will enable researchers without expertise in machine learning to train their own models without worrying about the intricacies of the underlying algorithms and networks.

### Parameter extraction

PyTrack extracts a total of 21 parameters related to the various eye movement events. These parameters have been selected after conducting a thorough survey of the most widely used parameters in the eye-tracking community. However, it is possible to add more parameters to the current list based on the requirements of the users.

### Data format support

Currently PyTrack offers support for EyeLink, SMI and Tobii devices as they are the most popular and widely used. However, there is scope for adding support for other eye trackers which are not commonly used or are relatively new in the industry.

### AOI

Currently, in order to supply multiple AOIs for a given stimulus, the analyze function of PyTrack has to be run multiple times. Although this is a relatively quick process, we plan on including the support for multiple and dynamic AOIs in the next release, in order to simplify the process.

### User Interface

PyTrack's visualization GUI is a convenient and easy to use interface but the analysis relies on basic coding. With that in mind, it is possible to convert the analysis component to a GUI also, thereby improving the user experience.

We believe that PyTrack can be integrated with ease into the analysis stage of an eye-tracking experiment to simplify the analysis process by providing a fully automated end-to-end pipeline that performs the necessary tasks of parameter extraction, statistical analysis and visualization. At the same time, PyTrack is flexible enough to allow users to access the internal workings and modify them according to their needs. As such, it is a solution that provides the dual functionality of (i) acting as a black box, thus removing complexities associated with computation; and (ii) acting as a white box, allowing the users to modify the pipeline at any stage to suit their needs.

The code, along with installation instructions and documentation, for PyTrack can be found at https://github.com/titoghose/PyTrack. Sample data has been provided to test the toolkit, which can be downloaded from https://osf.io/f9mey/files/. The SMI and Tobii files provided in the sample data have been obtained from the EYE-EEG toolbox (Dimigen, Sommer, Hohlfeld, Jacobs, & Kliegl, 2011).

#### Author note

We would like to thank Dr Dominique Makowski (School of Social Sciences, NTU, Singapore) for his helpful discussions and advice in developing PyTrack. We would also like to thank Nadine Garland for proofreading the manuscript. The support and assistance of Mr. Shivaprasad G (Dept. CSE, Manipal Institute of Technology), Dr. Jabez Christopher (Dept. of CSIS, BITS, Pilani - Hyderabad Campus), and Dr. Rishi Kumar (Dept. of Economics and Finance, BITS, Pilani - Hyderabad Campus) is greatly appreciated.

## Electronic supplementary material

Supplementary Fig. 1: Comparison of blink detection plots generated by (**a**) PyTrack and (**b**) Hershman’s Matlab code. The pairs of red circles mark the onset and offset of a blink. The data used to generate the plots are Trial 17 and 33 of the sample data provided by Hershman (https://osf.io/gjt8v/). (PDF 214 kb)

Supplementary Fig. 2: Comparison of microsaccade position-velocity plots generated by (**a**) PyTrack and (**b**) Engbert’s Microsaccade Toolbox for R. The plots generated are for the sample data file "f01.005.dat" provided with the toolbox. (PDF 72 kb)
