# Supplementary material for: PyTrack: An end-to-end analysis toolkit for eye tracking
Source: Behav Res Methods. 2020 Jun 4;52(6):2588–603. doi: 10.3758/s13428-020-01392-6 (PMC7725757; doi:10.3758/s13428-020-01392-6)
Supplement: Supplementary file 1 — Comparison of blink detection plots generated by (a) PyTrack and (b) Hershman’s Matlab code. The pairs of red circles mark the onset and offset of a blink. The data used to generate the plots are Trial 17 and 33 of the sample data provided by Hershman (https://osf.io/gjt8v/). (PDF 214 kb) [file 13428_2020_1392_MOESM1_ESM.pdf]

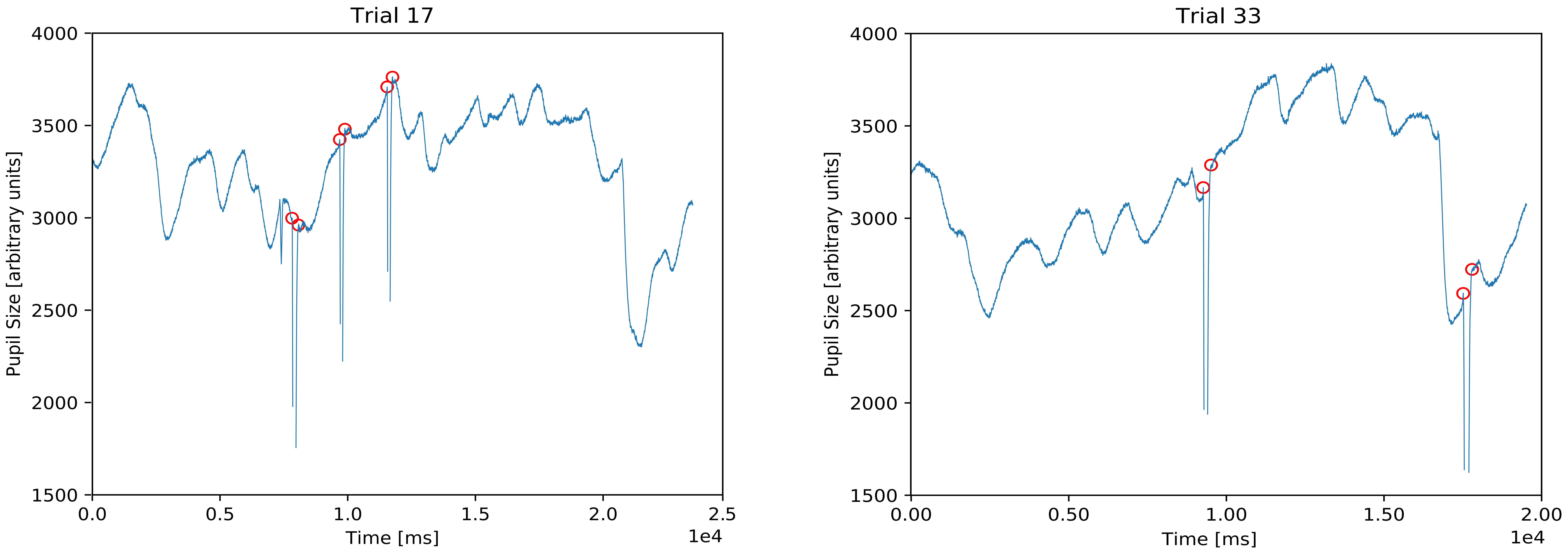

(a) Plots generated by the implementation used in PyTrack

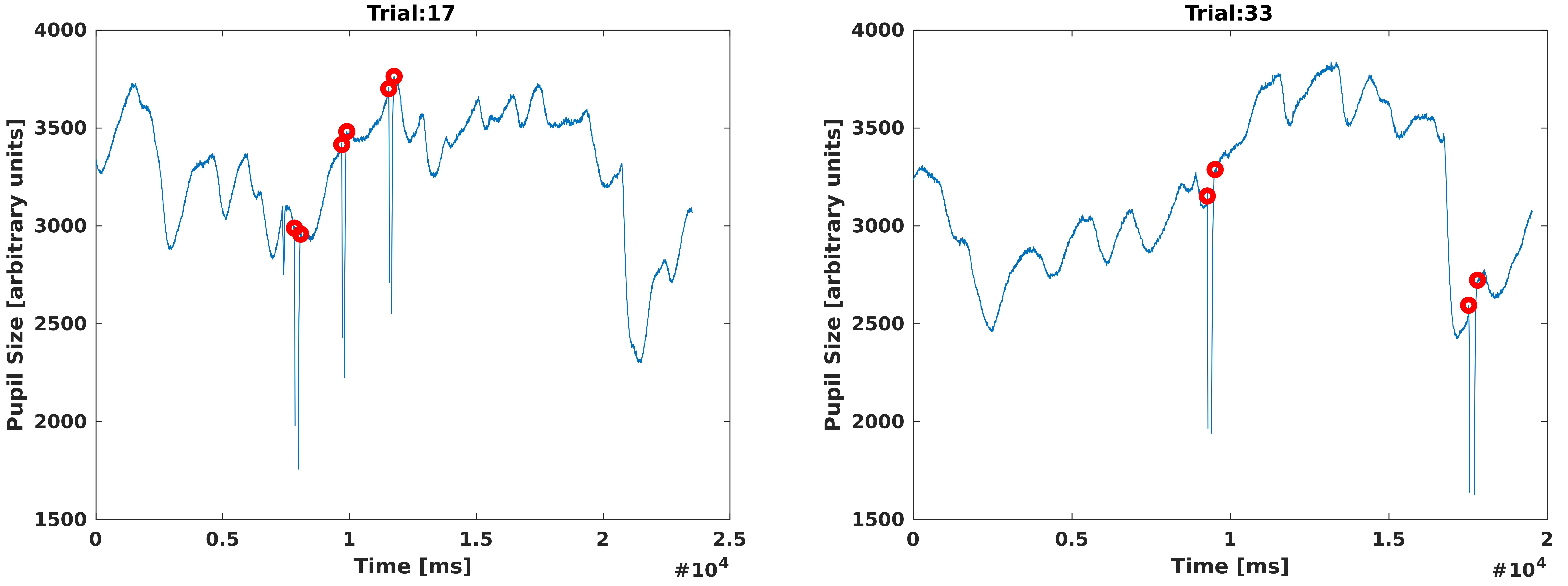

(b) Plots generated by Hershman's Matlab code
