# Supplementary figures and images for: PyTrack: An end-to-end analysis toolkit for eye tracking
Source: Behav Res Methods. 2020 Jun 4;52(6):2588–603. doi: 10.3758/s13428-020-01392-6 (PMC7725757; doi:10.3758/s13428-020-01392-6)

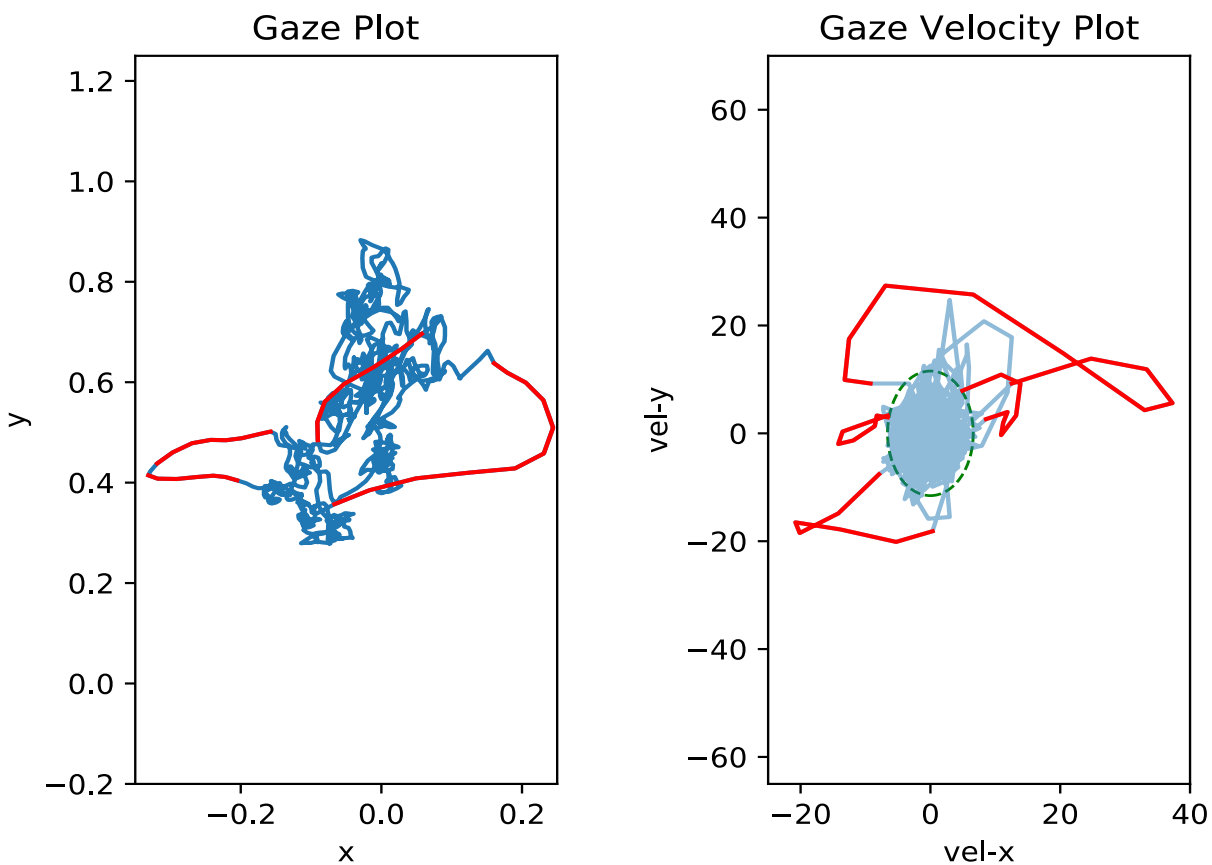

(a) Plot generated by PyTrack

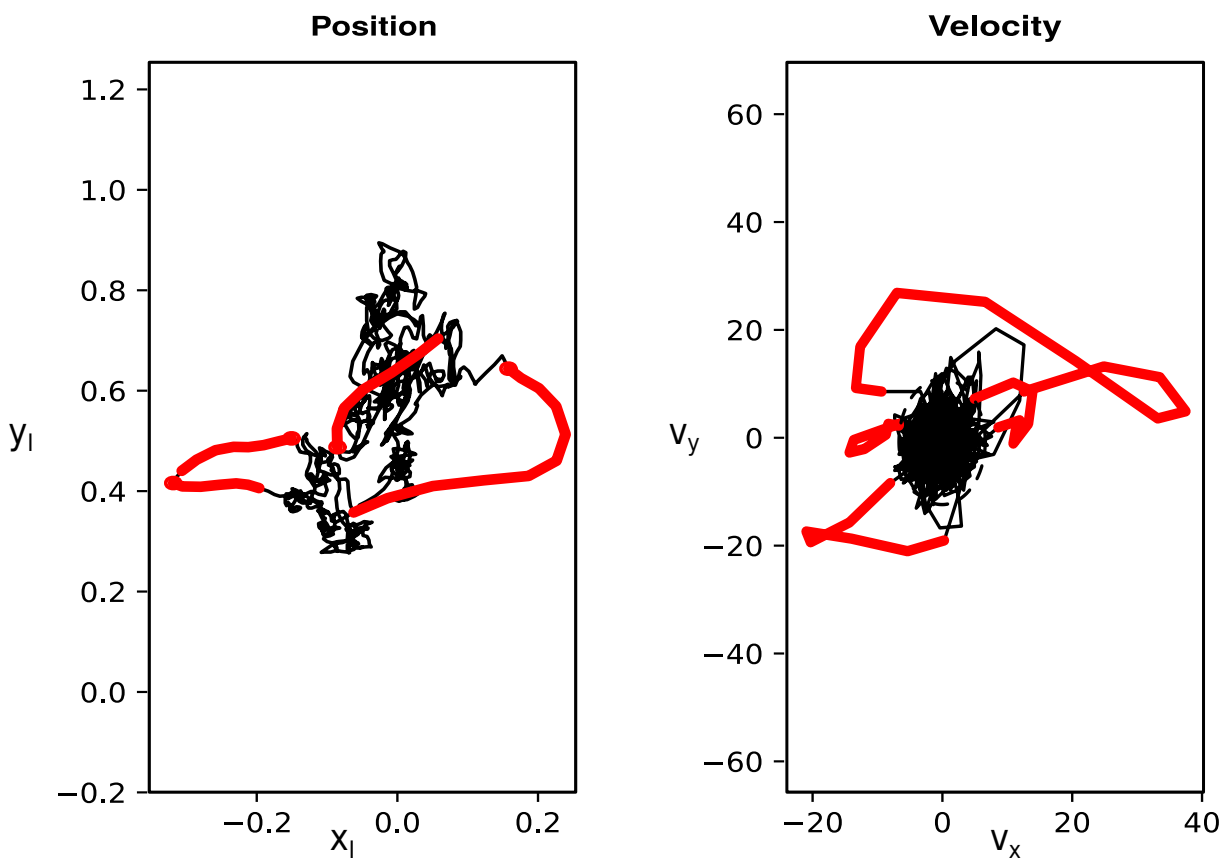

(b) Plot generated by Microsaccade Toolbox for R

Supplement: Supplementary file 2 — Comparison of microsaccade position-velocity plots generated by (a) PyTrack and (b) Engbert’s Microsaccade Toolbox for R. The plots generated are for the sample data file "f01.005.dat" provided with the toolbox. (PDF 72 kb) [file 13428_2020_1392_MOESM2_ESM.pdf]
